# Artificial Muscle Devices: Innovations and Prospects for Fecal Incontinence Treatment

**DOI:** 10.1007/s10439-016-1572-z

**Published:** 2016-02-29

**Authors:** Elisa Fattorini, Tobia Brusa, Christian Gingert, Simone E. Hieber, Vanessa Leung, Bekim Osmani, Marco D. Dominietto, Philippe Büchler, Franc Hetzer, Bert Müller

**Affiliations:** Department of Surgery and Orthopedics, Hospitals Schaffhausen, 8200 Schaffhausen, Switzerland; Biomaterials Science Center, University of Basel, 4123 Allschwil, Switzerland; Institute for Surgical Technology & Biomechanics, University of Bern, 3014 Bern, Switzerland; Department of Medicine, University of Witten/Herdecke, 58448 Witten, Germany

**Keywords:** Fecal sphincter, Electro-active polymer actuator, Biomimetic design

## Abstract

Fecal incontinence describes the involuntary loss of bowel content, which is responsible for stigmatization and social exclusion. It affects about 45% of retirement home residents and overall more than 12% of the adult population. Severe fecal incontinence can be treated by the implantation of an artificial sphincter. Currently available implants, however, are not part of everyday surgery due to long-term re-operation rates of 95% and definitive explantation rates of 40%. Such figures suggest that the implants fail to reproduce the capabilities of the natural sphincter. This article reviews the artificial sphincters on the market and under development, presents their physical principles of operation and critically analyzes their performance. We highlight the geometrical and mechanical parameters crucial for the design of an artificial fecal sphincter and propose more advanced mechanisms of action for a biomimetic device with sensory feedback. Dielectric electro-active polymer actuators are especially attractive because of their versatility, response time, reaction forces, and energy consumption. The availability of such technology will enable fast pressure adaption comparable to the natural feedback mechanism, so that tissue atrophy and erosion can be avoided while maintaining continence during daily activities.

## Introduction

The current aging of society has led to the increasing prevalence of social and economic burdening by age-related diseases. Among them is the loss of control of the defecation process denominated as fecal incontinence (FI).^34^ FI describes the involuntary loss of bowel content including flatus, mucus, liquid and solid feces. Severe consequences affect the individuals involved, i.e., exclusion from social life, isolation, and stigmatization.^34^ It has a considerable, but underestimated, economic impact.^57^

The overall prevalence of FI in adults is between 11 and 15% and increases with age.^34^,^57^ Stoker *et al.* reported that approximately one third of people living in retirement homes or similar institutions are affected.^57^ In U.S. retirement homes, FI prevalence is about 45%,^10^,^41^ which has been confirmed in a recent European cross-sectional study.^54^ Due to the under-reported nature of FI, it is likely that its true prevalence is even higher.^54^

Fecal continence relies on a complex interplay of the central, peripheral, and autonomous nervous systems, a functioning gastrointestinal (GI) tract, and the anal sphincter complex. A dysfunction of only one of these components can cause severe FI.

The ultimate goal of therapy is a subjective improvement of symptoms that increases the individual’s quality of life. If a patient’s first encounters with FI are without severe comorbidities, a conservative therapy attempt is advisable. Quite frequently, a change in sphincter pressure is not measured, but patients report fewer episodes of stool loss solely through life-style changes such as dietetics or after a period of pelvic floor training with biofeedback.

If conservative therapy is not successful or pelvic floor incidents are present, surgical therapy may be advisable. The extent of surgery depends on the severity of FI and/or sphincter lesion and compliance and/or patient age. In severe cases, artificial bowel sphincter systems may be an option. Systems used today are fluid-filled cuffs, which are so far not part of routine surgery due to many complications, such as wound infections or post-operative pain and consecutive re-surgeries. Unsuccessfully treated, severe forms of FI leave no other choice than the creation of a stoma. With a stoma, patients may gain a high amount of quality-of-life, but especially in young and active patients this option is not what colo-proctologists aim for.^29^ However, the controlled voiding of fecal matter in a pouching system *via* stoma opening is often a better solution compared to involuntary stool loss from anus.

**Figure 1:**
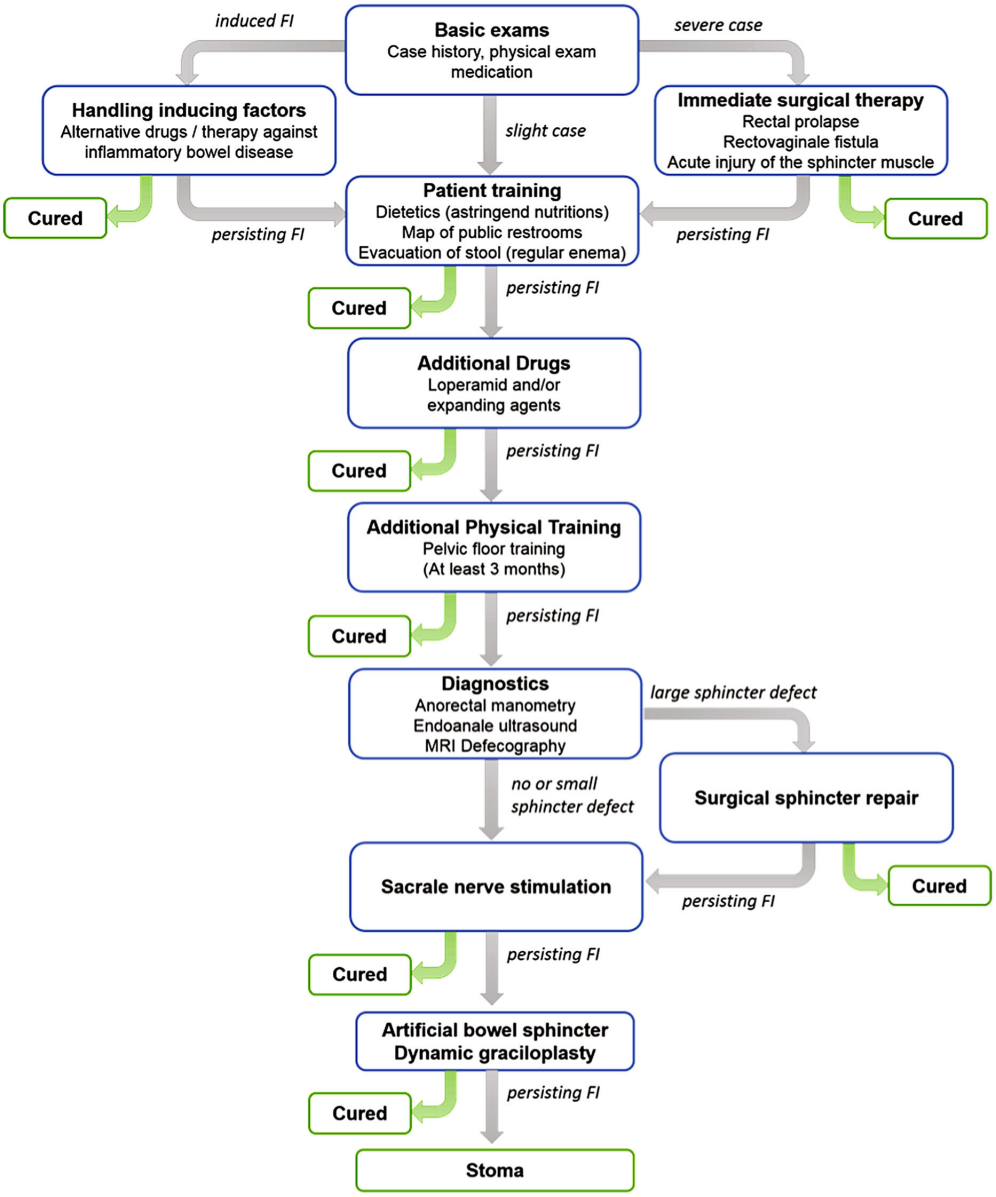
The flow chart shows the wide variety of the more or less complex FI treatments. If the conservative therapies fail, i.e., dietetics, medication, and pelvic floor training or pelvic floor incidents are present, a surgical approach is often advisable, which includes sphincter plastics, graciloplasty, and sacral nerve stimulation. In severe cases of FI, the artificial bowel sphincter systems can be applied. The ultima ratio is the creation of a stoma.

Figure 1 displays an overview of the treatment algorithm including modalities adapted from Gingert *et al.*^14^ In the end, however, there is no standard treatment. It seems that the optimized treatment is a complex combination of surgical and non-surgical therapies and is highly dependent on both surgeons’ and patients’ perception. Thus, treatment of stool incontinence belongs to specialized colo-proctologists.

Alternative developments in regenerative medicine aim to replace the sphincter with cultured human tissue functioning without a device. Tissue engineering has raised expectations, but the progress in growing muscle tissues artificially is slower than expected. Particularly, the preparation of blood vessels or nerves and their connections to ones of the patient remain a serious challenge. Furthermore, society has expressed ethical concerns about the use of stem cells and the *in vitro* construction of organs. Hence, the treatment of severe fecal incontinence based on regenerative medicine approaches is currently far from translation into clinical reality and, thus, not in the focus of the present review.^11^,^37^

## Overview of Systems to Treat Incontinence

The present review is based on intensive literature searches performed for scientific purposes in 2009 and 2012. In October 2014, the database PubMed was specifically screened using the keywords *artificial anal sphincter* and *artificial bowel sphincter*. From 455 hits, the 312 English abstracts of publications in English and German were considered. The reference list of the present review contains the more relevant publications for implant developments, which have been tested in animals and humans. The literature search has been repeated in December 2015 and resulted in 20 additional hits. These communications, however, were regarded as already covered or less relevant for this publication.

The review focuses on non-biological, active implants that replace the function of the continence organ. Devices, which require a partially functioning continence organ and reinforce the functionality of the sphincter muscles, are summarized together selected Refs. 8,9,36,48,49 in Table 1. The sacral nerve stimulation (SNS) has become a standard operative procedure for the treatment of fecal incontinence.^59^

**Table 1:** Currently available sphincter supporting implants i.e., bulking agents, slings, and sacral nerve stimulation, with details on material, working principle, outcomes, relevance and status. Clinical studies demonstrate limited success in the restoration of fecal incontinence.

	Material (examples)	Working principle	Outcomes	Relevance/Status
Bulking agents (injected or implanted materials incl. SphinKeeper™)	Collagen Autologous fat Silicone elastomers Teflon^©^ Ceramic-microspheres Polyacryloniterile^48^,^49^	Increases the volume around the anal canal, augment the passive closure pressure; self-expandable inter-sphincter prosthesis	Success rates: 23–95% over a 3–19 month observational period^36^ Success of placebo injections leads to success rates reaching 27%^36^	Improvement in symptoms at least in the first year Small sample sizes and lack of long term studies do not permit general conclusions about safety and effectiveness^36^
Slings (incl. FENIX^®^)	Fascia Tendon Silk Nylon Teflon^©^ Silicone	Anal encirclement by bands. Passive increase in pressure around the canal Elongates elastically, widens canal for rectal emptying	Complete continence without leakage: 18–75% Principal adverse events: Sling breakage, skin erosion/infection Total explantation rate 25–39%^8^,^9^	Lack of randomized controlled trials (RCTs) and long-term studies do not allow any recommendation to be made
	FENIX^®^: Titanium beads with magnetic cores linked by titanium wires	Anal encirclement by magnetic band Attractive magnetic forces between beads decrease with increasing bead separation	Clinical response^a^ is 0–9% Principal adverse events: pain, infection, bleeding, and fecal impaction	Investigational device Randomized clinical trials (RCT) started at end of 2013 in France and UK (estimated patient size of 156 and 250 patients)
Sacral nerve stimulation (SNS) or sacral neuromodulation		Low amplitude electrical impulses stimulate the sacral nerve, which activates pelvic floor musculature The exact mechanism underlying the improvement in continence achieved by SNS is actually unclear	Intention-to-treat success rate^b^: 63% in the short term^59^ In fact, the definitive SNS system will be implanted only if the test system improves continence of more than 50% Most common complications: Pain and paresthesia	Has become a standard operative procedure for the treatment of fecal incontinence Supported by medical committees and authorities worldwide

^a^ Clinical response = improvement of >50% in Cleveland Clinic Florida Fecal Incontinence Score

^b^ Intention-to-treat success rate = i.e., 50% improvement in incontinence episodes per week

**Figure 2:**
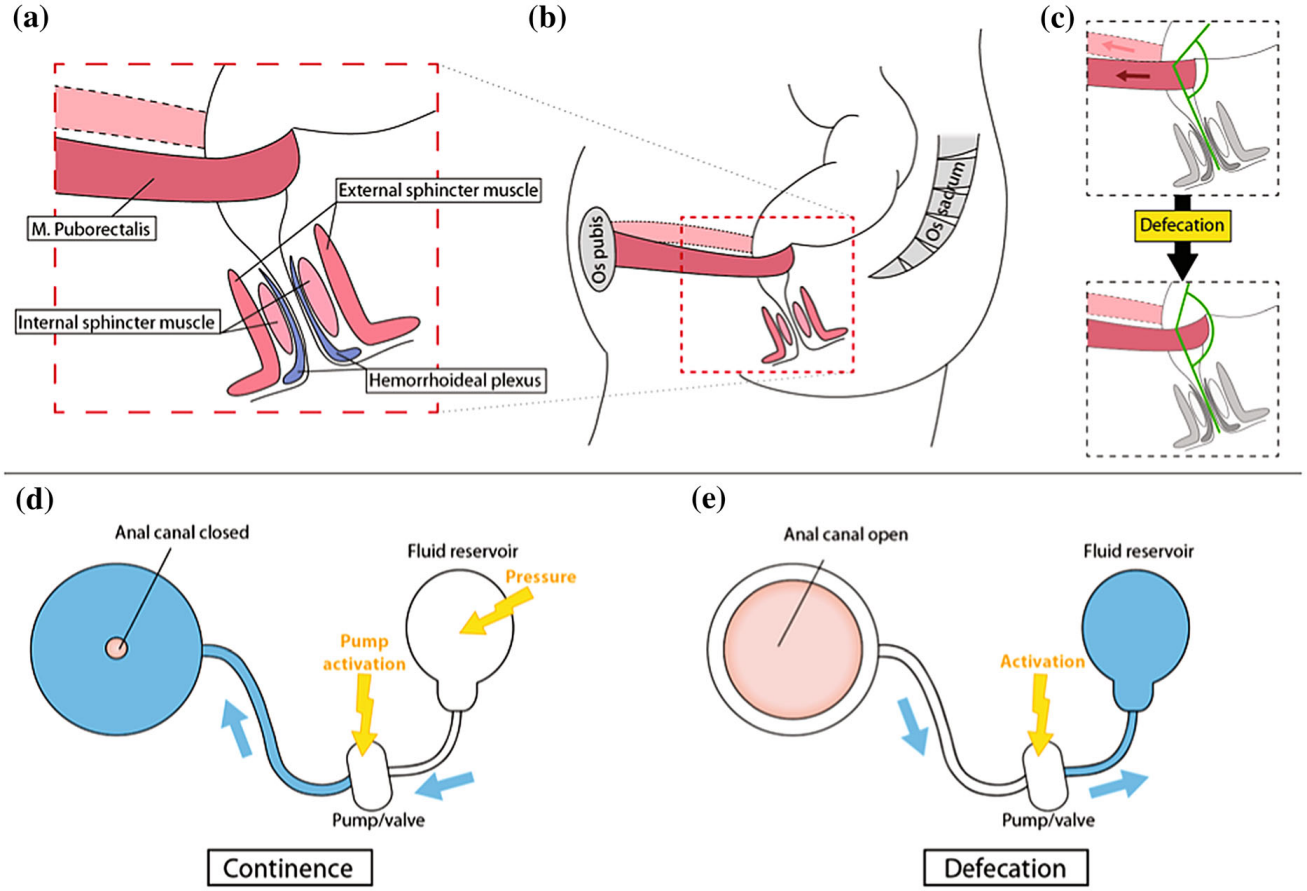
Opening in natural and fluid-cuff-based artificial sphincters. (a) Closeness depends on the intact continence organ consisting of an internal and external anal sphincter (IAS, EAS), hemorrhoid cushion, and puborectal muscle (*m. puborectalis*). (b) The puborectal muscle loops around the rectum and pulls it towards the *os pubis*, a ventral bone of the pelvis. (c) When the puborectal muscle is activated, the rectum is closed, and feces cannot descent from the rectal ampulla to the anal canal. For defecation, the puborectal muscle is relaxed, the rectum straightens, and faces decent. The relaxation changes the anorectal angle indicated by green color. The IAS, which is an involuntary muscle, relaxes as well by the reflex triggered by the distension of the rectal ampulla. If the EAS is voluntary relaxed, defecation is possible. (d) Currently, the artificial sphincters are generally based on fluid-filled cuffs. (e) Activating the pump to defecate, the fluid from the cuff is transported to the reservoir. To restore continence, the fluid is pushed back in the cuff as shown in (d). The majority of the FI systems are implanted around the EAS. The simple fluid-filled cuff systems constantly act on the underlying tissues. Because continence can only be ensured for rather high pressures, the tissues are compromised, which usually results in atrophy and erosion.

Figures 2a–2c schematically displays the anatomy and operation of the natural continence organ. Fluid-filled cuff devices as known from the treatment of severe urinary incontinence are most common today and work purely mechanically transferring fluid from cuff to reservoir to open the anal canal, cf. Figs. 2d–2e. The expanded cuff generates constant pressure acting onto the tissue almost the entire day. Within a relatively broad range the surgeon has to select the cuff pressure high enough to reach continence and low enough to avoid tissue damages including ischemia and erosion. It is important to note that the systems described in the literature essentially consist of a control module and an actuator.

Shape memory alloys (SMA) are materials, which provide shape changes suitable to switch from close to open stage of the anal canal. NiTi is especially interesting, as the transformation from martensite to austenite occurs at or a few Kelvin above body temperature.^39^ Thus, NiTi is used for medical applications including orthopedics, orthodontics, and cardiovascular treatments.^61^ Hence, one can find attempts to take advantage from SMAs for artificial sphincter devices.^1^ The challenge is to identify a design, which allows for switching within seconds with reasonable energy consumption.

A relatively simple approach to close and open the anal canal is the usage of (electro-)mechanics. For example, an electromotor can drive the mechanical clamp system^25^ or the elastic scaling cuff system.^28^ Here, the engineers have to build a system reliably working for decades with a reasonable energy transmission and consumption. Besides the rather conventional motors, one may apply dielectric elastomer actuators as recently proposed.^40^

The potential of tissue engineering (TE) to treat FI has been investigated with the aim to restore the function of the degenerated tissue using autologous cells.^6^ The main challenges are to replace the currently used, passive scaffolds to induce vascularization in constructs of centimeter size.^3^,^52^ If successful, tissue engineering using autologous cells represents a major medical breakthrough.

## Natural Continence and Assessment

The intact continence is difficult to understand, as multiple factors and interrelated mechanisms contribute. Injuries such as trauma from childbirth, psychological and neurological disorders, and inflammatory diseases of the bowel can cause the loss of continence control.^14^,^21^ Figure 2 schematically illustrates the main continence structures. The *puborectal sling* pulls the rectum towards the *os pubis* leading to the curved anatomy of the rectum. This shape combined with the closure mechanism containing three layers enables continence. The outer layer, comprising the *puborectal sling*, the *M. levator ani* and the circular external anal sphincter muscle (EAS), narrows the anal canal. The EAS is a voluntary muscle, which can multiply the pressure in the anal canal during contraction for a restricted period of time. The *pudendal nerve*, connected to the sacral root S3/4, innervates the EAS. The middle and internal layers involve the *M. sphincter ani internus* (IAS) and hemorrhoid vascular cushion and ensure the continence to gas and liquids. The parasympathetic fibers connected to the sacral cord and the sympathetic fibers starting from the lumbar cord innervate the IAS.^13^ The hemorrhoid vascular cushion contains arterial-venous vessels. The contraction of the venous vessels fills the cushion and completely closes the anal canal. Other mechanisms of continence, i.e., the anti-peristaltic function of the sigmoid, ensure that the rectum remain empty. Dedicated receptors within the rectum and anal canal can detect the presence and consistency of stool. The signals induce rectoanal reflexes and defecation cycles that allow for the complete evacuation of stool from rectum and anal canal.^13^

Diagnosis of the affected patients starts with the assessment of their frequency of defecation and the ability to hold back flatus and stool. As next step the medical expert searches for direct signs of incontinence including erythema, scars and smearing, which are instantly detected by inspection. The examination generally includes the rectal palpation, since divergent pressures due to lesions of the sphincter or irregularities in the structure of the anal canal could be present. Manometry serves for the quantification of the sphincter’s functionality. In some instances, medical experts not only use the conventional anal manometry but also the three-dimensional (3D) manometry with balloons to assess the rectal capacity and sensibility. Endoanal ultrasound (EUS) allows detecting the layers of the sphincter apparatus and the integrity of the entire sphincter system. The volume of defects including scars is easily assessable. Magnetic resonance imaging (MRI), and especially MR-defecography, is a further examination technique to testify dyssynergies and further pathologies of the pelvic floor by image acquisition during evacuation. This extensive and expansive examination is embarrassing for the patients and hence only applied to substantiate suspicion of complex pathologies of the pelvic floor.

## Clinically Available Artificial Sphincter Prostheses

### AMS 800 and Acticon™ Neosphincter

In 1987 the AMS 800™ from American Medical Systems, LLC, an existing artificial urinary sphincter system, was the first neo-sphincter applied to treat fecal incontinence. The fluid-filled cuff around the anal canal allows for continence, see Figs. 2d–2e. In order to release stool, one manually pumps the liquid from the cuff to the reservoir. It was necessary to adapt and refine this passive, purely mechanical implant system to the bowel anatomy. The result was the Acticon™ Neosphincter available since 1996 and FDA-approved in 2001. This device also contains a septum port to easily adjust the fluid quantity after implantation.^2^ The implantation includes three to four incisions to place the cuff around the anal canal, the reservoir to the abdomen and the 12 mm-wide and 36 mm-long pump into the scrotum or labium in a subcutaneous pouch.

A systematic review on safety and effectiveness of the Acticon™ Neosphincter reports revision rates and definitive explantation rates of 94 and 39% pooled proportions for the 5-year follow-up, respectively.^22^ The indications for surgical revision, covering 535 patients, were device malfunction (36%), tissue erosion (29%), infection (28%), re-implantations (12%), and pain (10%).^22^ The reasons for the definitive device explantation were mainly infection (56%), erosion (37%), and device malfunction (19%). Besides the adverse events, solid stool continence has been achieved in 96, 98 and 63% of the patients at short-, middle-, and long-term, respectively.^22^ Continence to liquids varied between 71% at short term and 45% at long term, whereas continence to gas was only achieved in 47 and 11% of the patients at short- and long-term, respectively.^22^ Hence, this relatively simple implant only has limited success, which significantly decreases with increasing time from implantation.

### Prosthetic Anal System (PAS)

**Figure 3:**
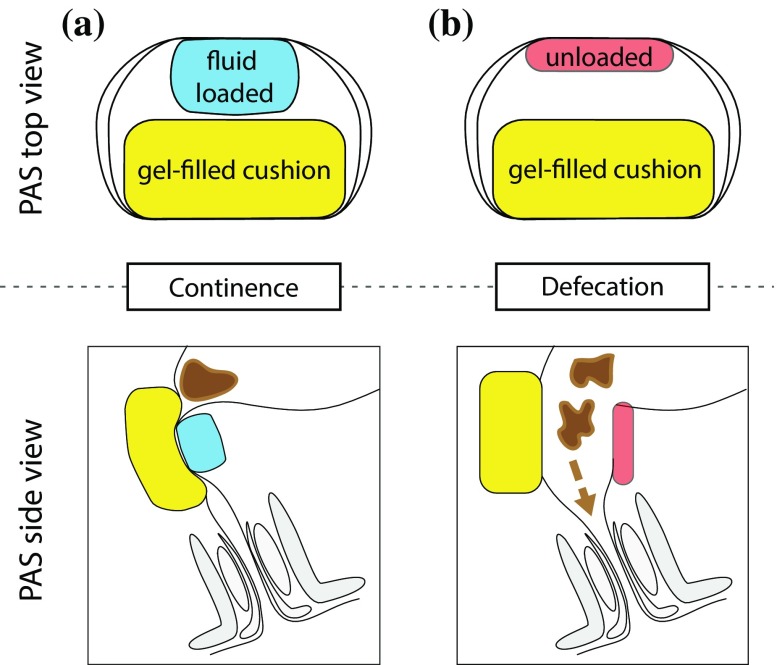
Scheme of the PAS in (a) close and (b) open states. As the other fluid-filled cuff systems, the PAS works through mechanical obstruction. Similar to the puborectal muscle, cf. Fig. 2(c), the PAS increases the angle of the anorectal junction to gain continence. The PAS also contains a gel-filled cushion, given in yellow, which deforms, when the pressure in fluid-filled cuff is changing. The PAS is placed above the sphincter around the rectum close to the anorectal junction implying many advantages. First, there is much more space and less vulnerable structures than close to the anus. Second, the minimally invasive surgery for the PAS placement is comparably easy and safe, which results in a relatively low infection rate and the absence of extended scar tissue.

In 1992, the prosthetic anal system (PAS), another fluid-based system, was introduced. It is schematically represented in Fig. 3. An asymmetric sphincter element is placed around the anorectal junction. In order to angulate the bowel, the fluid-inflatable linear element acts against the soft gel-filled pillow. The inflation of the linear element induces the bending of the gel-filled pillow and the tissue enclosed. This bending reduces the pressure required to ensure continence.^17^ The patient manually opens and closes the sphincter element.^12^

In detail, straps connect the linear element and gel pillow forming a lumen with a mean diameter of 22.5 mm.^19^,^20^ The system operates at the pressure plateau, i.e., the pressure in the regulating balloon reservoir is kept constant.^12^ The pressure transmission from expander to intestinal lumen is about 60%.^19^,^20^ The working range of the plateau pressure corresponds to values between 10 and 13 kPa, i.e., 72–100 mmHg.^19^

The sphincter element is placed around the anorectal junction in the abdominal cavity and implanted *via* a lower midline abdominal incision with the linear expanding element dorsally and the gel pillow ventrally to the anorectal junction. Thus, the pressure profile on the bowel resembles a hammock distribution and removes the crenation effects of the circular devices. As a consequence, continence to solid feces is achieved at operating pressures as low as 5 kPa, i.e., 40 mmHg. Experiments on eleven patients using Doppler scanners have proven that the related blood flow reduction is only 30% and ischemic injuries are avoided.^20^

A mini-pig study demonstrated the pros and cons of the PAS and allowed for significant improvements.^18^ The subsequent clinical study with twelve patients showed limited success.^12^ One major postoperative complication led to device removal. Device removal was also required in two other patients due to infection and device separation. At later stage another patient needed a revision surgery because of device separation. Furthermore, pump unreliability resulted in revision surgery of four patients. Nevertheless, continence after one year was stated in ten of eleven patients (91%). All patients suffered from constipation. They needed aperients and enemas. Two patients even required a bowel washout in hospital.^12^

### A.M.I.^®^: Soft Anal Band

A.M.I.^®^ Soft Anal Band has also been a fluid-based system for continence treatments in use since 2005. Pressing the valve placed in a subcutaneous abdominal pouch opens the cuff. Its closing requires manual activation by squeezing the fluid reservoir. Therefore, the patient can manually control the cuff pressure. At rest, for example, a significantly reduced pressure improves tissue perfusion and regeneration.^4^ In addition, the pressure-regulation port allows the amount of fluid to regulate the maximum exerting pressure. An intraluminal closure pressure of 9 kPa, i.e., 66 mmHg, is reached with 24 mL saline solution.^15^ The operation of the system through the skin is still tricky especially for elderly people despite modifications by the manufacturer.^15^ The neo-sphincter is implanted through four incisions. Two perineal incisions are needed to introduce the soft anal band cuff. A low-abdominal incision serves for the passage of tubing from pelvic to abdominal cavity plus one incision in the abdominal wall for the implantation of valve, reservoir, and septum port.

More than 200 patients obtained the A.M.I.^®^ Soft Anal Band, and first reports on the outcome have become available.^15^ The preliminary data presented in a conference abstract^45^ is supported by a clinical trial with 43 patients. The authors report complication, revision and explantation rates of 48.8, 32.6 and 21.0%, respectively.^15^ The adverse events following device implantation reported are wound infection with 9%, penetration with 2%, and intolerable pain with 2%. Two patients could not actuate the device themselves and required help from close relatives.^15^ Although the Soft Anal Band^®^ efficiently improves continence, these incomplete results show that there is relatively high complication, revision, and explantation rates as well as the rather difficult handling, which prevented a broader application of the device.

## Current Research on Artificial Sphincter Prostheses

### Shape Memory Alloy Sphincter

Heating and cooling shape memory alloys (SMA) can reversibly change their shape as the result of martensite-austenite phase transformation. The appropriate choice of the Ni–Ti ratio in SMA gives rise to a transformation at body temperature or slightly above, see for example Ref. 47 Therefore, such a mechanism could become the basis of a powerful sphincter system. In 2001, the first SMA-based artificial anal sphincter device was proposed.^1^ This device contains two SMA plates connected by hinges. A direct current through the plates induces a temperature increase that results in the austenite transformation between 47 and 52 °C. Heat flow through the human tissue reduces the temperature associated with the martensite transformation between 49.5 and 44.5 °C.^32^

The system operation is rather complicated, since the patient has to open the anal canal using transcutaneous energy transfer (TET) for inducing the resistive heating. To avoid thermal damages of the surrounding tissue and to allow healing after the surgery, a hinge mechanism keeps the canal open for the necessary periods of time.^31^

The SMA plates, covered by silicone for thermal isolation, are 65 to 75 mm long, 10 to 15 mm wide, and 0.5 to 0.7 mm thick. Considering the thickness of the silicone layers, 3 mm on the outer side and 4 mm on the luminal one, yields a lumen of about 30 mm. Because 10 of these 30 mm are used for silicone pillows, the space for the intestinal tissue and stool passage results in 20 mm.^32^

The implantation in animals was performed creating an end-colostomy through the abdominal oblique muscles, fixing the device around the stoma to the intestines with latches, and placing the device *via* extra-peritoneal approach between abdominal wall and peritoneum.^30^ A one-month animal study with a Landrace piglet was performed applying the insulated and heat-protected artificial sphincter device.^42^ The sphincters were actuated three to four times a day, in total 105 times. The autopsies revealed (1) infection around the device, (2) burns around the secondary coil of the TET system, and (3) a small quantity of stool accumulated in the colon. A series of one to three months studies with goats was performed to test safety and efficacy of the pre-clinical prototype.^31^ The authors had not detected any sign for infection around the implant but found a 3 to 5 mm-thick fibrous capsule around all components after 13 weeks. This capsule presumably influenced the force acting on the tissue, which was originally set to 5 to 8 kPa, i.e., 40 to 60 mmHg. In the preliminary tests, the safety and efficacy of the system could be reached for a period of three month in a sheep model. No communication on clinical trials has been found, although the promising animal studies are more than 10 years old.

In 2004, the same research team proposed another more compact and simpler SMA device to replace or support the function of the puborectal sling.^33^ This curved beam is fixed on the coccyx and pushes to close the canal. Activation straightens the beam and hence opens the anal canal. The proposed function, based on the tissue resistance, is difficult to verify.

### German Artificial Sphincter System (GASS)

The German Artificial Sphincter System (GASS) is based on a fluid-filled cuff with the main components compactly combined around two supporting rings hinged at an angle of 180°. The two fluid reservoirs are fixed on the outer diameter of the supporting ring, whereas the multi-chamber occluding polyurethane part is placed inside the ring. The fluid can be transported from the outer to the inner chambers and vice versa by the bidirectional, piezo-technology-based micro-pump, which, together the electronics, has a size of 83 × 45 × 27 mm^3^.^53^ Its miniaturized control unit is subcutaneously placed through laparoscopic implantation. The research team developed a bidirectional pump with reasonable energy consumption and emergency capability for cuff opening.^55^ The development of the GASS III prototype included a reduction of the actuation voltage from 320 to 30 V, a fluid velocity of 2.23 mL per minute, which corresponds to a period of eight to nine minutes for the complete transport of the fluid volume, and a four-day charging cycle for the battery assuming three defecations per day. The patient actuates the sphincter *via* remote control.^53^

For the mini-pig study, the cuff was implanted around the lower rectum in a botulin toxin-induced incontinent animal *via* a perianal incision.^55^ No communication on clinical trials has been found, although the promising *in vitro* tests with porcine anal canals are more than ten years old.^55^ The most recent communication on the GASS was published in 2010 and reported a redesign by separating the occluding cuff from the fluid reservoir and pump. Further GASS development seems to be ongoing together with an industrial partner.

### Artificial Anal Sphincter System (AASS)

The compression unit of the Artificial Anal Sphincter System (AASS) is based either on a set of fluid-filled cuffs^63^ operated by means of a micro-pump with a motor gear,^26^ or a mechanical clamp unit^25^ actuated *via* the electrification of an electromagnet that pushes the two hinged metal plates apart,^25^ or an elastic scaling cuff driven by a micro-motor that retracts and loosen the steel wire rope within the elastic mechanism.^28^ It further comprises a sensory part and a control unit with rechargeable battery and radiofrequency antenna. The patient operates the sphincter *via* remote control and recharges the battery *via* TET.

Pressure sensors^27^ or the infra-red (IR) sensor^23^ tell the patient they need to defecate. When the pressure reaches the predefined threshold, the patient gets informed. After the completion of rectal voiding the pressure falls below another predefined threshold and a related signal is displayed on the remote control.^23^ The thresholding, however, needs signal processing of the pressure by filtering and subsequent wavelet package analysis and support vector machine to detect a meaningful indicator for the need to defecate.^62^ Besides the pressure sensors, the IR sensor detects the presence of feces in the rectum, which, however, only reliably works *in vitro*.^23^

The implant of the most recent AASS has a total volume of 55 × 45 × 25 mm^3^.^26^ It was implanted in one mini-pig dissecting the lower rectum. The fluid-filled cuff was positioned around the end of the rectum with subsequent enteroenterostomy. The TET coil was subcutaneously placed near the groin. The anal sphincters were removed to ensure incontinence in the animal.^26^ After 13 days the pressure sensor failed. The wireless communication was stable and reliable. The TET system was effective. There was no infection, but the presence of a fibrous capsule was found.^26^

The fluid-filled cuff system with the IR sensor was tested in eight rabbits with limited success.^23^

The AASS system has to be refined before successful clinical studies could start.

## Comparing the Artificial Sphincters for FI Treatment

Table 2 provides an overview of the artificial sphincter systems on the market and in development. It contains the relevant information including operation principle, applied pressures, response time, geometry, surgery procedure, and implant location.

**Table 2:** Overview of the currently available artificial sphincter systems (Acticon^®^ Neosphincter, PAS, Soft Anal Band) and the prototypes under development (GASS, AASS, SMA).

Characteristics	Acticon^®^ Neosphincter^2^,^22^	PAS^12^,^18^,^19^	Soft Anal Band (AAS)^15^	GASS^53^,^55^	AASS^26^	AS-SMA^1^,^31^,^33^
Operating pressure (mmHg)	60–66, 67–74, 74–81	60–70, 40^a^	66^a^	24–58^ab^	30–54^ab^	40–60^ab^
Opening/closing time	–/5–8 min	–/(Design 2002) 5 min	–/–	8–9 min^c^/8–9 min^c^	80 s/80 s	<1 min/–
Compression cuff						
Dimensions	∅ 29–45 mm Width 20–29 mm	Linear element: 55 × 25 × 17 mm^3^ (open) 55 × 25 × 36 mm^3^ (closed) Cushion: 110 × 25 × 17 mm^3^ Open cuff ∅ 22.5 mm	Small/medium/large (With two extensions)	∅ 65 mm (open) ∅ 40 mm (closed) Width 20 mm	∅ 30 mm Width 25 mm	Length 80 mm Width 15 mm Height 20 mm Open cuff ∅ 20 mm
Material	Silicone elastomer	Silicone	Silicone	Polyurethane	Polyethylene and silicone	NiTi and silicone
Surgical implantation	Perineal	Lower-middle abdominal incision	Perineal	Perineal^b^ (Targeted laparoscopic)	Rectal anastomosis^b^	End colostomy^b^
Location of components	Scrotum or labium, prevesical space	Subcutaneous pouch at iliac fossa, peritoneal cavity	Subcutaneous pouches above iliac crest and abdomen	Below abdominal wall^b^	Subcutaneous pouches in the abdomen and groin^b^	Subcutaneous pouch^b^
Working principle	Fluid-filled cuff	Fluid-filled cuff	Fluid-filled cuff	Fluid-filled cuff	Fluid-filled cuff	Shape memory alloy
Scheme of cuff (top view)						
Open						
Closed						

Device specifications such as operating pressure, opening/closing time, cuff size, material, implantation method, component location, working principle and geometry are compared

– not available

^a^ Intraluminal pressure

^b^ In animals (*in vivo* and/or *in vitro*)

^c^ 16–18 mL at ca. 2 mL/min

The commercially available artificial sphincter muscles are fluid-driven mechanical systems to be manually operated by the patient. The patients are generally elderly people with limited fine motor skills and force generation. The actuation through the skin enhances tissue response to the implant materials, trigger inflammatory body responses, and consequently cause device malfunction.^58^ It can also result in migration and perforation of the subcutaneous pouch culminating in extrusion from the skin.^50^ The AMS and PAS systems require multiple squeezes of the pump to completely open the compression cuff without feedback about the filling status of the cuff, which could result in ineffective cuff voiding, retaining the fecal matter in the rectum.

The time to open and close the sphincter depends on the construction of the occlusion cuff and on the system pressure, but is much longer than for the natural counterpart. It corresponds to more than five minutes for the closure of the AMS as well as the first PAS^18^ and reaches nine minutes for the GASS.^53^

## Physical Principles for the Operation of Artificial Sphincters

**Figure 4:**
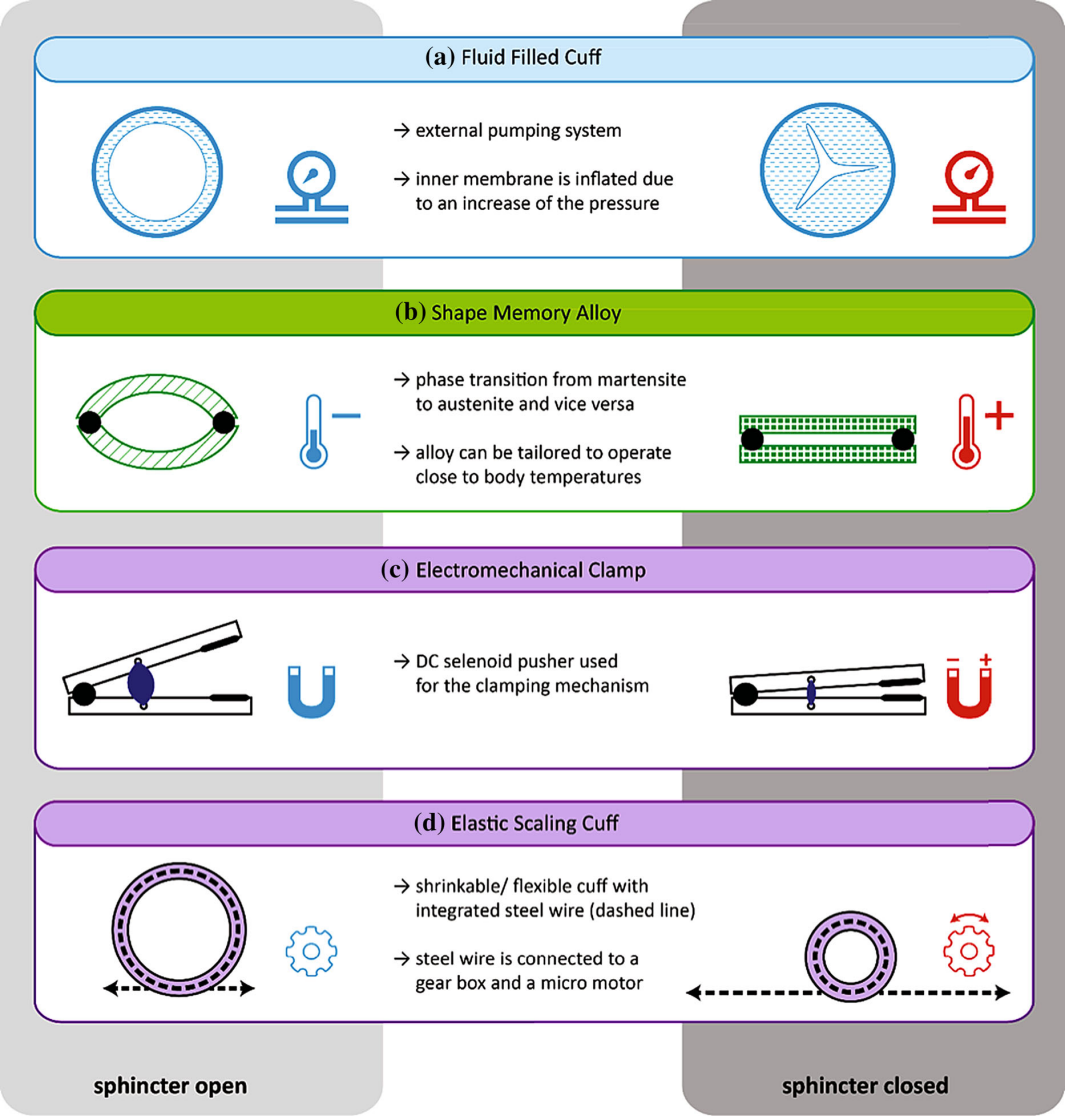
Schematic representation of the actuation principles currently used for commercially available artificial sphincters and systems under development. (a) An inflatable membrane of a fluid-filled cuff squeezes the tissues according to the induced pressure. (b) Two plates made from the shape memory alloy Ni–Ti deform as the result of a temperature increase to 55 °C. Thermal isolation is required to avoid the heating of the surrounding tissues above 43 °C. (c) An electromagnet switches the hinged clamp mechanism. The silicone rubber housing has to be carefully designed to ensure secured gripping. (d) The elastic scaling cuff contains a circularly stretchable mechanism with an integrated steel wire rope. The gearbox connected to a micro-motor rolls up the wire resulting in contraction. The springs are encapsulated in silicone.

**Figure 5:**
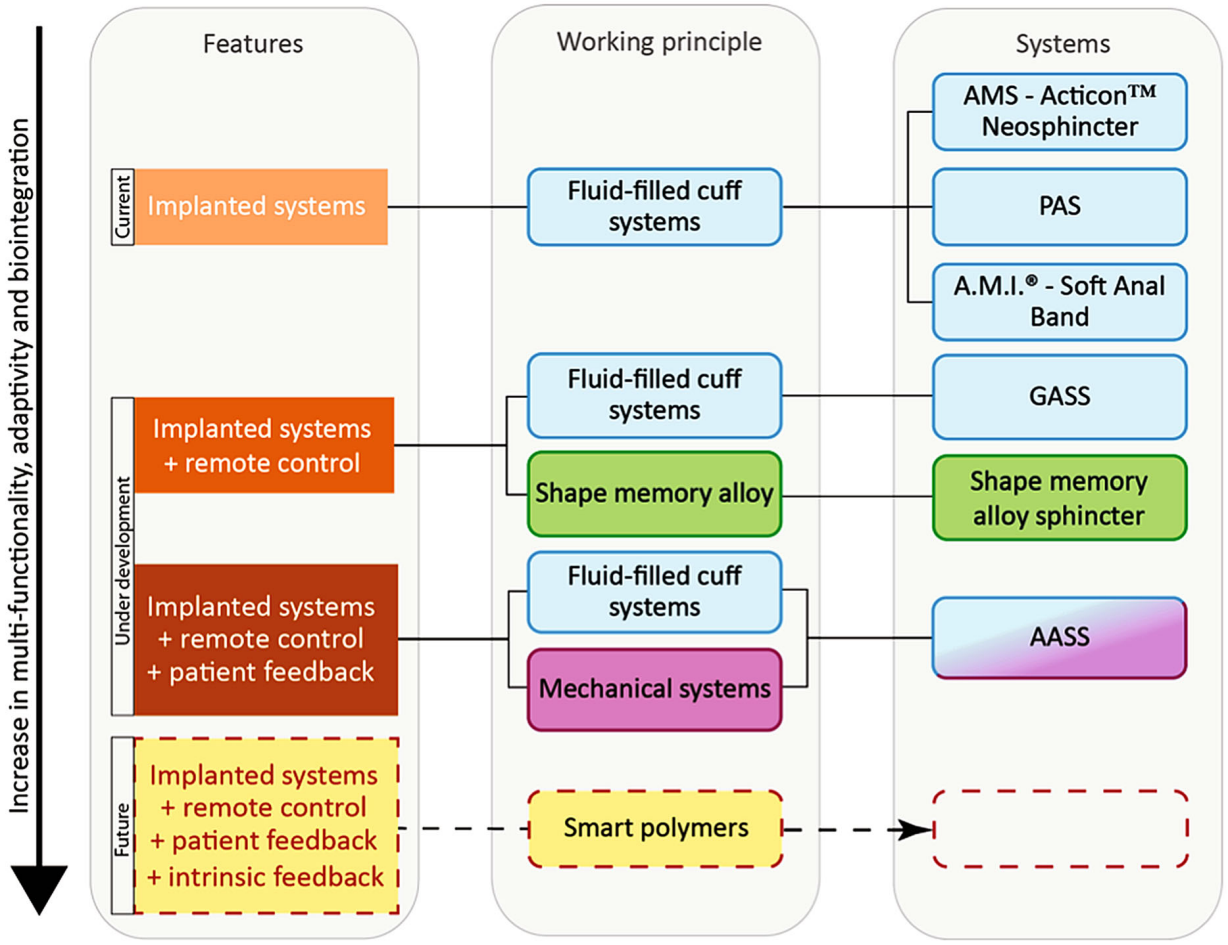
Roadmap of approaches toward breakthrough in non-biological artificial sphincter development. Active sphincter devices to replace the function of the continence organ include electromagnetic, shape memory alloy-based, and, in particular, fluid-filled cuff systems. The diagram shows from top to bottom the currently commercially available implants, the prototypes currently in animal studies, and the systems under development for future artificial sphincters. The columns list the features of the individual systems, the related physical principles of operation and the system’s denomination. The Acticon™ Neosphincter is the only device approved by the Food and Drug Administration (FDA) so far. The Soft Anal Band is clinically available as investigational device. The Prosthetic Anal System (PAS) has also been implanted in humans. For the German Artificial Sphincter System (GASS) and the shape memory alloy sphincter reports on *in vitro* and animal studies are available. A Swiss consortium, the SmartSphincter team of nano-tera.ch, develops a low-voltage, dielectric actuator sphincter with sensory feedback. AMS: American Medical Systems, A.M.I.: Agency for Medical Innovations, AASS: Artificial Anal Sphincter System.

Figure 4 summarizes the physical mechanisms for opening and closing of the prototypes in use. In the simplest case, one can use a manually driven mechanical pump to inflate a cuff that surrounds the anal canal. Such a pressure can also be generated using the thermally activated SMA components, the mechanical clamping by means of an electromagnet, or a gear connected with a flexible cuff. The experience with the currently implanted devices (AMS, PAS, Soft Anal Band), however, clearly elucidates that the incorporation of further components into the entire sphincter system is necessary, cp. Fig. 5. The GASS and the SMA sphincter take advantage of a remote control, which includes electronics, a wireless control unit, and TET. The application of wireless communication and energy transmission requires the compliance with safety regulations. The regular adaptation of the artificial sphincter by the patient and/or the responsible medical expert *via* feedback, as integrated into the AASS, is another feature towards mimicking of natural continence. Up to now, no system is realized that uses intrinsic feedback, i.e., the closure pressure acting on the anal canal is adapted to the smallest possible pressure to guarantee continence.

## Alternative Principles for the Operation of Artificial Sphincters

### Embedded Pneumatic Networks

Recently, the research team of G. Whitesides developed embedded pneumatic networks (EPNs) that enable actuation in soft elastomers by pressurizing embedded channels.^24^ They use networks of channels in elastomers that inflate like balloons for actuation, which exhibit a strain large enough for an artificial muscle device. Their design and actuation control allow complex movements and closing mechanisms. The actuation rates, however, might be too small for sphincter applications. Furthermore, the encapsulation as necessary for the artificial sphincter is a remaining challenge.

### Electro-thermal Actuators

Baughman’s fishing line and sewing threads belong to the electro-thermally driven actuators as alternatives for artificial muscles.^16^ A straight, sub-millimeter-thick nylon fiber is heavily twisted until coils develop. The spring-like polymer coils have specific power densities up to 100 times larger than human muscles and strains as large as 34% within the temperature range from 20 to 240 °C. Silver coatings around the polymer fiber improve the actuation control due to direct electrical heating. It is, however, questionable, whether the necessary strains of about 10% can be realized at body temperature. The low energy consumptions could boost this technology for future implant applications.

### Ionic Electro-active Polymers

Ionic electro-active polymers consisting of a polymer electrolyte sandwiched between two metal electrodes have been proposed for soft machines for the last two decades.^7^,^35^ Voltages in the range of 1 to 4 V displace the ions of the polymer film causing swelling and shrinkage, which can result in twisting, rolling, turning, and other non-symmetric bending depending on electrode design. The energy efficiency is generally less than 2%.^35^ Stresses of 30 MPa have been reported.^56^ So far, the application potential is restricted due to the actuation speed and the strains of only about 3%.^56^ More recently, swellable polymers, i.e., highly stretchable hydrogels, were introduced. Often they are weak and brittle but researchers from Japan have designed such hydrogels with polyrotaxane cross-linkers that are extremely stretchable and mechanically sufficiently strong.^5^ Mechanical forces, pH value, temperature, and electric field affect the swelling.^38^ Since the actuation relies on the ion diffusion, the reaction time is too long for the artificial sphincter.

### Dielectric Elastomer Actuators

Dielectric electro-active polymers were proposed for biomimetic actuation.^44^ These actuators show millisecond response time, mechanical strains of several 10%,^46^ and a specific continuous power up to 10 times higher than human skeletal muscles.^35^,^46^ They consist of incompressible but deformable elastomer films sandwiched between compliant electrodes. The elastic polymer film transduces the electrical energy into mechanical work by shrinking in thickness and expanding in area. Common micrometer-thick polymer films require actuation voltages close to the kV-range to obtain the necessary strain level for artificial sphincters.^44^ The necessary forces with physiologically acceptable voltages can be realized fabricating stacked polymer films of nanometer-thickness.^40^ Additionally, electrodes have to be stretchable to follow about 10% strain without significant changes in conductivity.^51^ Once these challenges have been mastered, this actuation principle is more than promising for the realization of artificial sphincters. Single-layer dielectric actuators that show the desired properties have very recently been fabricated by means of vacuum deposition.^60^

## Conclusion for the Next-Generation Device

The experience shows that using a constant pressure for closure has serious limits. As for the natural sphincter the adaptation to the bowel pressure changes, which are significantly influenced by the physical activity, and non-compromising of the tissue is a prerequisite for future devices as well as the long-term performance of the systems. The necessary forces cannot be adapted manually, since the patient is not fast enough and mechanical manipulation through the skin has many drawbacks. Therefore, a sensory feedback and a sufficiently fast actuator with relatively low energy consumption will form the core of a future artificial anal sphincter.

The implantation procedure has to be simple and safe to avoid infection for the non-sterile conditions in the perineal area. Currently, placement of the components requires more than one incision. Consequently, the surgeon opens the body with two or more skin incisions, related to high risk of infection and patient distress. Future devices should become rather smaller than larger compared to the size of the available systems, see Table 2.

One can also expect strong impact on the device design by the significantly improved understanding of continence in health and disease. In these regards, realistic computational models of biomechanical processes of the involved anatomical structures can guide the development of patient-specific treatments.^43^ The review identifies the device requirements and some issues to be considered for the design, summarized in Table 3.

**Table 3:** Basic requirements for the design of the optimized artificial anal sphincter in terms of patient’s anatomy, device performance, and safety constraints.

	Constraints	Requirements
Anatomy	Compression unit: Shape and dimensions	Personalized/selected to patient’s anatomy
	Components (battery, telemetric device)	
	Location in the abdomen	Single subcutaneous compact pouch
	Dimensions	Maximal 50 mm × 50 mm × 4 mm
	Connections between components	Wired inside the body
Performance	Avoid tissue erosion/atrophy	Cyclic reduction of pressure for tissue regeneration
	Pressure profile	Adapted to physical activity *via* ms sensory feedback
	Strain of actuator	About 10%
	User-controlled opening	Telemetric control as simple as possible
	Voiding time	Less than 15 min for full cycle
	Recharging time	About 15 min
	Opening and closing time	Each less than 1 min
	Durability	20 years
Safety	Biocompatibility	According to ISO 10993
	Operating voltage	24 V or less
	Temperature at tissue-device interface	36–37 °C
	Emergency system	Two redundant systems
	Failure emergency system	Manual opening by patient possible

We are confident that based on their versatility, reaction speed, reaction forces, as well as energy consumption, smart materials such as low-voltage dielectric elastomers can be used to produce artificial muscles. This technology, together with a consideration of tissue reaction to the implant, changes in the properties of surrounding tissues as well as device miniaturization and compactness in the design of the device will lead to an optimized artificial anal sphincter implant. We have to state, however, that this approach is a reasonable option to explore, but should not be overestimated until further evidence becomes available.
